# A Novel Method to Couple Electrophysiological Measurements and Fluorescence Imaging of Suspended Lipid Membranes: The Example of T5 Bacteriophage DNA Ejection

**DOI:** 10.1371/journal.pone.0084376

**Published:** 2013-12-23

**Authors:** Nicolas Chiaruttini, Lucienne Letellier, Virgile Viasnoff

**Affiliations:** 1 ESPCI Paristech, CNRS, Paris, France; 2 Aurélien Roux Lab, Biochemistry Department, University of Geneva, Geneva, Switzerland; 3 Institut de Biochimie et Biophysique Moléculaire et Cellulaire, Université Paris Sud-11, CNRS, Orsay, France; 4 MechanoBiology Institute of Singapore, Singapore, Singapore; Université d'Evry val d'Essonne, France

## Abstract

We present an innovative method to couple electrophysiological measurements with fluorescence imaging of functionalized suspended bilayers. Our method combines several advantages: it is well suited to study transmembrane proteins that are difficult to incorporate in suspended bilayers, it allows single molecule resolution both in terms of electrophysiological measurements and fluorescence imaging, and it enables mechanical stimulations of the membrane. The approach comprises of two steps: first the reconstitution of membrane proteins in giant unilamellar vesicles; then the formation of a suspended bilayer spanning a 5 to 15 micron-wide aperture that can be visualized by high NA microscope objectives. We exemplified how the technique can be used to detect in real time the translocation of T5 DNA across the bilayer during its ejection from the bacteriophage capsid.

## Introduction

Electrophysiological measurement of ion channel activity plays an important role in deciphering biological processes since the advent of the patch clamp method [1]. In particular, reconstitution of purified membrane proteins in lipid bilayers is used to study mechanosensitive ion channels as well as the molecular mechanism of ion transport and of ion channel inhibition by drugs. In the past decades it has also been used as a tool to study DNA translocation across membranes through artificial or proteinaceous pores [2] that were used as molecular sieves [3] or sensors [4]. Recent advances in microfabrication now allow the commercialization of planar systems to automatically form suspended bilayers or to perform patch clamp experiment ([5], Oxford Nanopore Technologies, Nanion Technologies). However, most of these approaches are not compatible with simultaneous detection of electrophysiological signals and high resolution imaging. On the one hand, suspended bilayers are usually the tool of choice when transmembrane ionic current is to be measured. On the other hand, supported bilayers are preferred for quality imaging. Coupling both measurements is of interest to decipher complex mechanisms that lead to proper ion channel electrical activity, such as dimerization [6,7]. Furthermore, the interpretation of current traces alone can be ambiguous at it is bound to electrically active events only. In this respect, the coupling with optical detection is of particular interest in the study of viral infection where several electrically inactive events (virus binding, protein conformational changes) are required prior to, or after, DNA translocation [8–13]. A few approaches were devised that allow simultaneous detection of both electrical and fluorescence signal [6,14–16]. However, very few can allow single molecule or single virus resolution owing mostly to the difficulty of stably forming bilayers, measuring conductivity and controlling transmembrane hydrostatic pressure in a device compatible with high resolution imaging.

In this paper we propose an alternative approach that consists in reconstituting the membrane proteins in a giant unilamellar vesicle (GUV) before bursting it open over a 5-15 µm wide aperture at the tip of a glass capillary. Single ion channel detection was achieved. Membrane tension was regulated by setting the transmembrane hydrostatic pressure, while high resolution imaging could be performed by moving the capillary tube a few microns away from the observation chamber bottom. In this paper, we demonstrate how this approach can be used to follow the various steps leading to genomic DNA ejection of the T5 bacteriophage. A T5 bacteriophage is constituted of a 100 nm proteinaceous capsid filled with a 127 kb-long genomic DNA. The capsid is linked to a 200 nm-long tail. *In vivo*, the DNA is ejected into the bacteria after the tail has bound to a transmembrane receptor protein (FhuA) on the surface of the bacteria.

## Materials and Methods

Bacteriophages: T5st(0), a heat stable deletion mutant (114 kbp, Genbank Acc AY692264) was produced on the host strain *E. coli* F and purified on cesium chloride gradients as previously described [17]. Phages were stored in phage buffer (10 mM Tris HCl pH 7.6, 100 mM NaCl, 1 mM MgSO_4_, 1 mM CaCl_2_) at 4°C. The final titers were 1.10^13^ pfu/mL. Phages were stained by incubating them 2 hours in phage buffer supplemented with an intercalating fluorescent dye (Yo-Pro I, Life technologies ref n°Y3603) at 1 µM. In these conditions Yo-Pro I diffuses through the capsid to label phage DNA. Staining did not result in significant loss of the phage ability to eject its DNA. If phages are not preincubated, Yo-Pro I weakly labels intracapsid DNA, but strongly and instantaneously stains DNA as soon as it is expelled from the capsid. 

FhuA: The gene encoding the outer membrane protein FhuA was overexpressed in *E. Coli* HO830 transformed with plasmid pHX405, and the protein was purified by using the previously described protocol [17]. FhuA was stored at a concentration of 50 µM in 20 mM Tris-HCl buffer pH 7.8, containing 250 mM NaCl, and 1% Octyl-β-D-glucopyranoside (OG).

FhuA labeling: labeling was performed with Alexa Fluor 568 carboxylic acid, succinimidyl ester (Life technologies ref n°A2003). Before labeling, Tris was removed from the stock solution by microdialyzing 100 µL of FhuA (QuixSep, CelluSep membranes MWCO = 3.5 kDa) in a buffer containing 20 mM Hepes pH 8.3, 250 mM NaCl, and 1% OG. Labeling reaction was done by mixing FhuA with Alexa (1 mg/mL) during 1 hour. The solution was then thoroughly dialyzed in buffer Hepes 20 mM pH 8.3, NaCl 100 mM, LDAO 0.06% (lauryl dodecyl amine oxide). Labeled FhuA molecules were recovered at 5 µM and their activity on T5 phages was nearly unchanged (Figure S1).

Reconstitution of FhuA in GUVs: We adapted the method used by Girard et al. [18] to incorporate FhuA in GUVs. First, small unilamellar vesicles (SUVs) were produced by drying 1 mg of DPhPC lipids (1,2-diphytanoyl-sn-glycero-3-phosphocholine, Avanti Polar Lipids ref n°850356P) on the surface of a glass test tube. The pellet was then suspended by vortexing in 100 µL of phage buffer, creating a cloudy solution. 5 µL of OG at 20% w/v was added, resulting in a clear solution. Detergent-purified FhuA (stock solution or fluorescently labeled) was incubated for 1 hour in the mix to reach a final weight ratio of proteins to lipids of 1/100. This solution was then dialyzed in a microdialyzer (QuixSep, CelluSep membranes MWCO = 3.5 kg/mol) against phage buffer. At the end of dialysis, the solution was cloudy again indicating the formation of proteoliposomes with incorporated FhuA. GUVs were subsequently produced by drying 0.5 µL droplets of the FhuA proteoliposomes on ITO coated glass slides (Sigma Aldrich, ref n°639303-1EA). The droplets were left in a humid salt saturated chamber (70% humidity) for 30 minutes. ITO slides were then paired together to form a sealed chamber filled with a solution of Sucrose (220 mM) + NaCl 1 mM matching the osmolarity of the phage buffer (~220 mOsm). GUVs were then electroformed by applying an AC field (2 V, 10 Hz) for 2 hours at room temperature. Imaging: all experiments were performed on an inverted microscope (Axioplan; Carl Zeiss, Jena, Germany), with a 63x water objective (NA 1.2). The fluorescence illumination source was a 120-W UVICO 2000 lamp (Rapp Opto-Electronic, Hamburg, Germany). Images were acquired by an iXon EMCCD camera (Andor Technology, Belfast, Northern Ireland). Temperature was set at 25°C. 

Fabrication of capillary pipettes: a capillary tube (Polymicro, TSP0XX150, 150 μm outer diameter (OD), where XX = 05, 10, 15, 20 correspond to available internal diameters (ID)) was held with appropriate connectors (Idex: F-330 (Nut, Fingertight), F-238 (Tubing sleeve, Microtight), P-659 (Adapter)), into a homemade micromanipulation device. The capillary was centered and introduced over 1 mm into the smallest end of a cone pipette tip (2-200 µL, Eppendorf). The plastic tip was subsequently melted onto the capillary tube to ensure adequate electric seal. The capillary tube was then cut with a diamond knife as close as possible to the cone tip leaving a clean flat cleaved glass surface onto which GUVs spontaneously fused. Capillary pipettes could be batch-processed and kept for weeks. The typical access resistances of those pipettes in phage buffer were: R(ID = 20 μm) = 3 MΩ, R(ID = 10 μm) = 10 MΩ, R(ID = 5 μm) = 40 MΩ.

Pipette micromanipulation: A capillary pipette was filled with approximately 100 µL of phage buffer then attached perpendicularly to a microscopy chamber. It was held with a micromanipulation device that allows to move the pipette in x, y, z directions and to correct for tilt angles (+-3 degrees) in order to put the capillary section parallel to the glass slide (Newport V100-CP). We used interference fringes between the pipette tip and the bottom coverslip of the microscopy chamber to ensure an optimum parallelism of both surfaces. Voltage control was realized using Ag/AgCl electrodes connected to an Axopatch 200B patch clamp amplifier (Molecular Devices, Sunnyvale, United States). The cis compartment (outside the pipette) was grounded. The electrical signal was filtered at 1 kHz and digitized at 2 kHz. Phage activity was monitored using a bias of -20 mV. Inner (trans compartment) pressure was also controlled by connecting the pipette to a pressure control system (MFCS-VAC-4N -25 mbar, Fluigent, Villejuif, France) that sets pressure within the pipette with fast response (< 0.1 s) and ~25 µbar accuracy. This control was used to counterbalance capillarity and gravity effects as well as to regulate lipid membrane tension.

## Results

The phage receptor protein FhuA is notoriously difficult to reconstitute into lipid bilayers using classical techniques [8,19]. Since the inactivated state of the protein is not an ion channel, protein insertion cannot be followed in time. Additionally, it is a very hydrophobic protein prone to rapid aggregation and denaturation in aqueous solvents. The most reliable reconstitution technique was obtained in small unilamellar vesicles [20]. Our first step in order to achieve the reconstitution of FhuA in large suspended bilayers was to insert this protein into GUVs.

### Reconstitution of FhuA into GUVs

**Figure 1:**
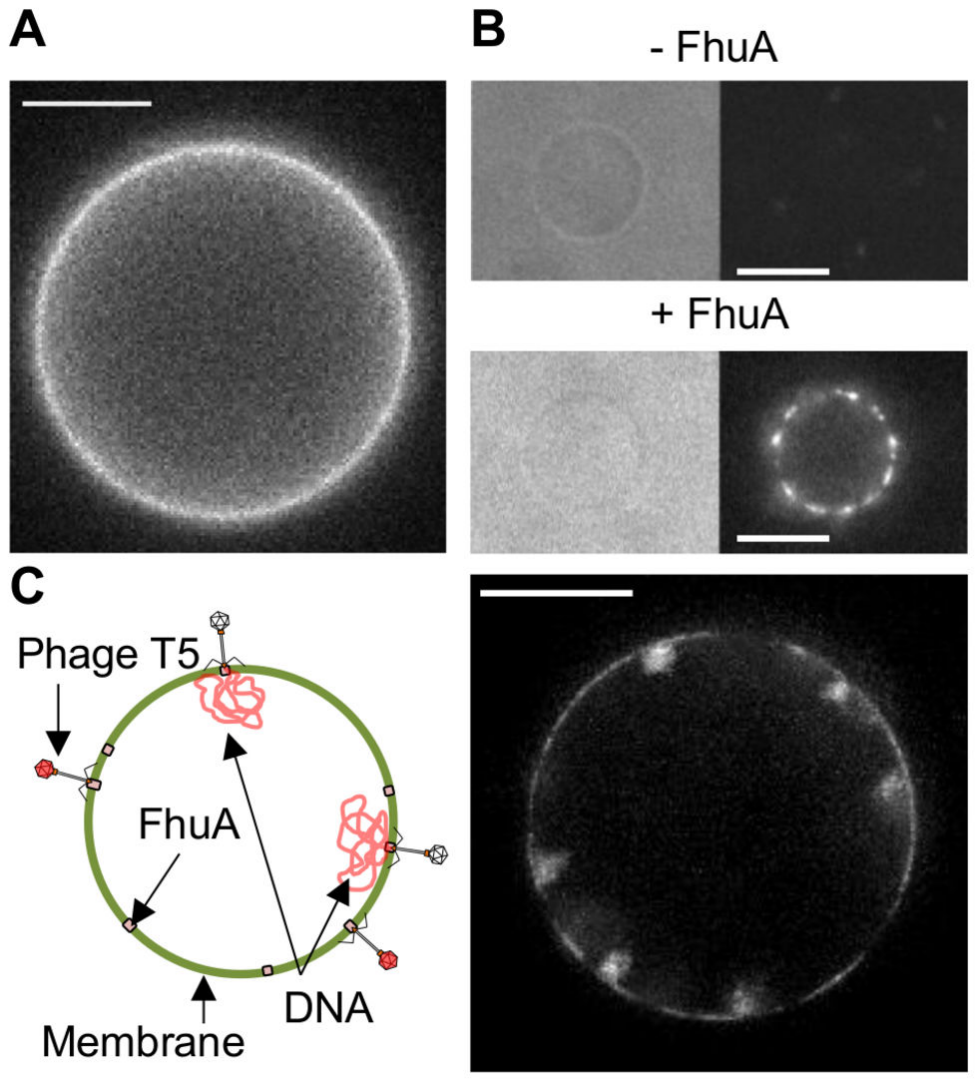
T5 DNA ejection process reconstituted in GUVs. A\ Fluorescence image of a GUV reconstituted at a DPhPC/FhuA-Alexa568 ratio of 100 w/w. B\ Combined brightfield (left) and Yo-Pro I fluorescence image (right) of DPhPC GUVs with FhuA (+FhuA) or without FhuA (-FhuA). Fluorescent dots correspond to individual phage particles stained with Yo-Pro I. Phages are either unbound (-FhuA, diffusing rapidly in solution: low fluorescence intensity) or bound to the vesicle surface (+FhuA, slow diffusion of phages on the vesicle surface: high fluorescence intensity). C\ Left: Schematic representation of bound phages that have released or not their intracapsid DNA onto the vesicle lumen. Right: Yo-Pro I fluorescence image of a DPhPC/FhuA GUV (100 w/w ratio) after 20 minutes of incubation with T5 phages (10^10^ pfu/mL). The image is a maximum intensity projection over time (30 frames taken at 10 fps). The time projection emphasizes the difference between a sharp fluorescence coming from the vesicle surface and the ‘cloudy’ fluorescence coming from the vesicle lumen. The sharp fluorescence corresponds to weakly stained phages bound to the vesicle that have not ejected their DNA. The ‘cloudy’ fluorescence comes from DNA molecules that have crossed the membrane, after their ejection by phages. 6 highly fluctuating individual DNA molecules can be distinguished, which remain attached at the vesicle periphery. All scale bars: 10 μm.

We adapted the protocol previously described in [18] to create GUVs functionalized with fluorescently labeled FhuA. GUVs were formed by electroswelling partially dried SUV pellets. Figure 1A displays the GUV homogeneous fluorescence, illustrating the efficient incorporation of FhuA, which did not aggregate during the process. The potential loss of receptor functionality was then tested. GUVs were placed in an observation chamber at 25°C. Phages were pre-labeled with a DNA intercalating Dye (Yo-Pro I) that, under appropriate conditions, can stain the intracapsid DNA (see material and methods). Phages were subsequently added at a final concentration of 10^10^ pfu/mL. They rapidly localized (~1 min) at the GUV membrane with no detected unbinding events. After a typical lag time of 10 minutes at 25°C, DNA molecules were present in the lumen of the GUV (Figure 1C). In the control (no reconstituted FhuA) only transient reversible adsorption events (Figure 1B) were observed. These results demonstrate that a fraction of FhuA is still functional after reconstitution, with an activation kinetic compatible with other *in vitro* studies [9,21,22].

### Patching GUVs to form large suspended membranes

**Figure 2:**
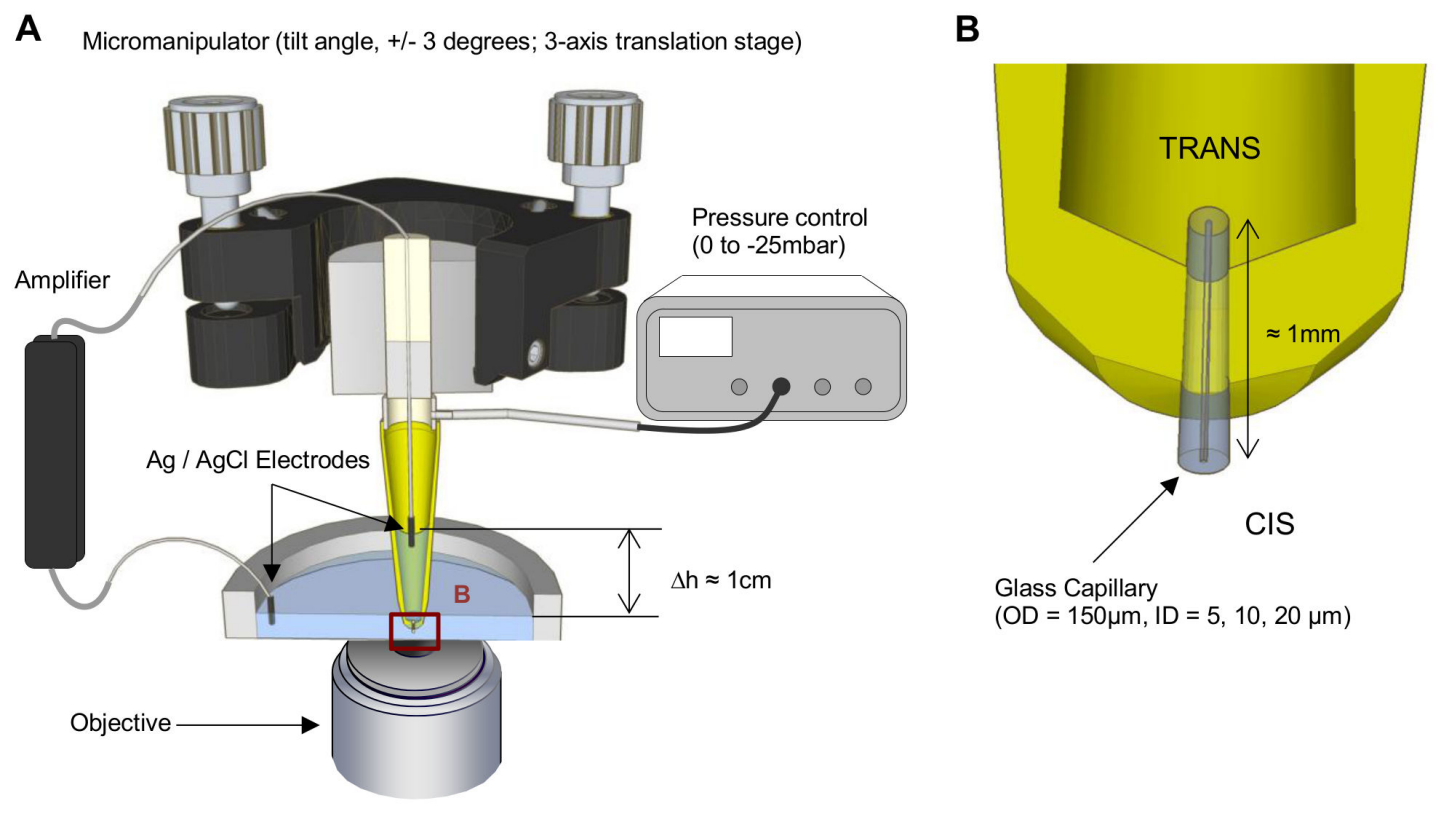
Capillary patch clamp setup. A\ Overview of the setup showing the chamber and the capillary pipette with associated voltage control (Axopatch 200-B), pressure control (MFCS) and micromanipulation devices (Newport). The pipette is cut in half for display purpose. B\ Zoom on the tip of the capillary pipette showing the inserted slice of capillary connecting cis and trans compartment. OD/ID: Outer/Inner Diameter.

To perform simultaneous transmembrane conductance measurements with optical detection, a single GUV was burst open on the tip of a cleaved capillary tube with a high ratio between outer diameter (OD) and inner diameter (ID). This ensures an efficient electrical seal while allowing optical detection by a high numerical aperture objective. We proceeded as follows (see materials and methods for details): we used commercially available capillary tubes (Polymicro) with an ID ranging from 2 to 40 µm and an OD ranging from 90 to 150 µm. We chose capillaries with an OD of 150 µm and an ID of 10 µm as a good compromise between imaging and the necessity of obtaining a large enough GUV. The capillary was introduced in a pipette tip as shown on Figure 2B. The polymer tip was melted around the tube to ensure adequate electrical insulation. The tube was then cleaved with a diamond cut roughly 1 mm from the pipette tip in order to ensure minimal access resistance at the membrane. 

The pipette was mounted on a micromanipulator and placed in the observation chamber (Figure 2A) over the objective. Equilibration of hydrostatic pressure between cis and trans compartment was performed using a pressure control system. It is a critical step to ensure stable vesicle fusion.

**Figure 3:**
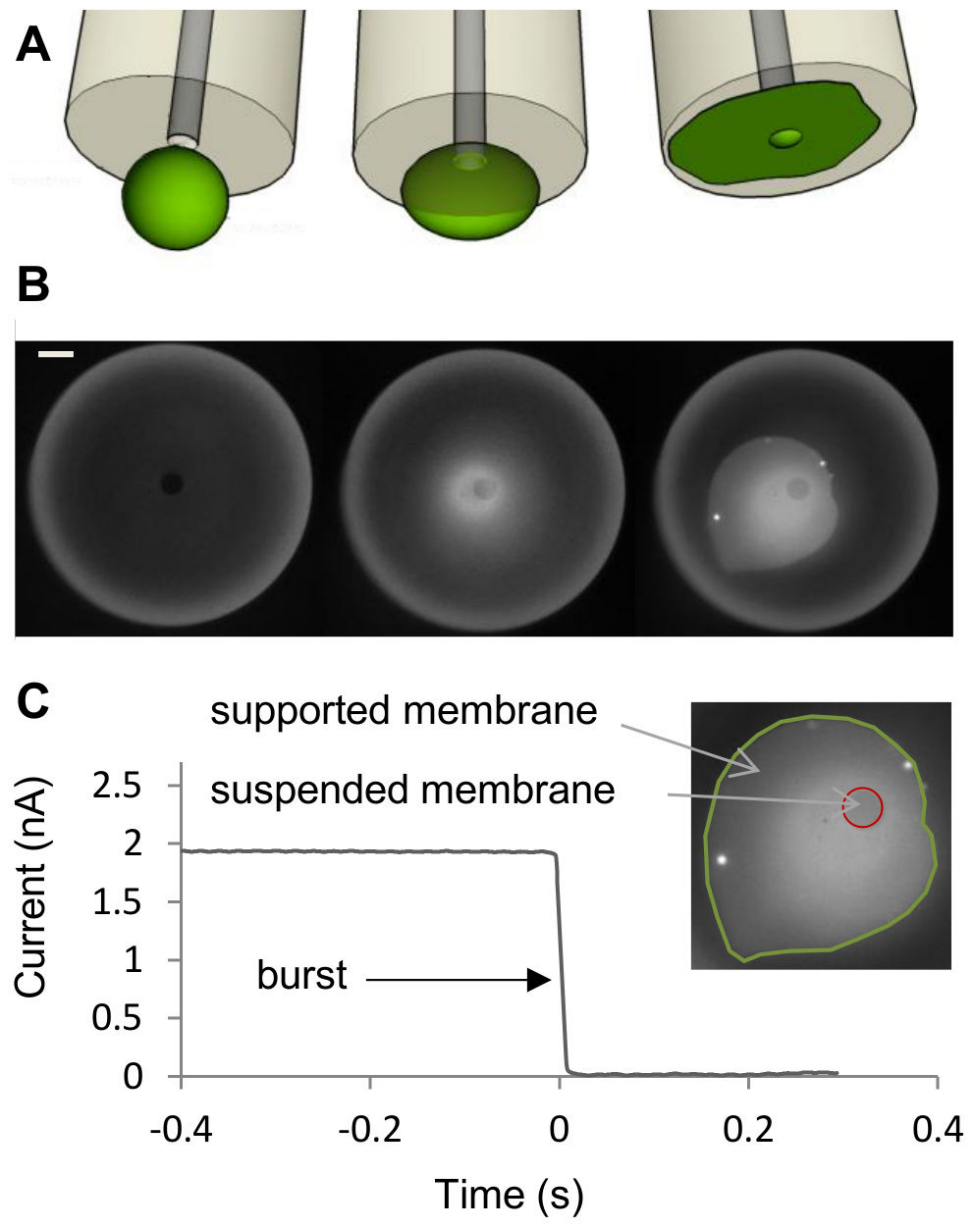
GUV bursting on capillary pipette tip. A and B\ Fluorescence images depicting the steps for GUV patch formation. The glass capillary tip is constantly maintained in focus. The DPhPC GUV is fluorescently labeled with FhuA-Alexa568 molecules. From left to right: the GUV is raised progressively until its top reaches the pipette tip (middle). The GUV bursts on the capillary glass section (right). Scale bar = 20 μm. C\ It leads to an immediate current drop that is monitored by maintaining a constant voltage through the pipette (-20 mV). A seal is obtained if the membrane completely covers the internal pipette diameter, delimited by a red circle in the inset. This suspended membrane is connected to a supported membrane, which is delimited by a green line in the inset.

**Figure 4:**
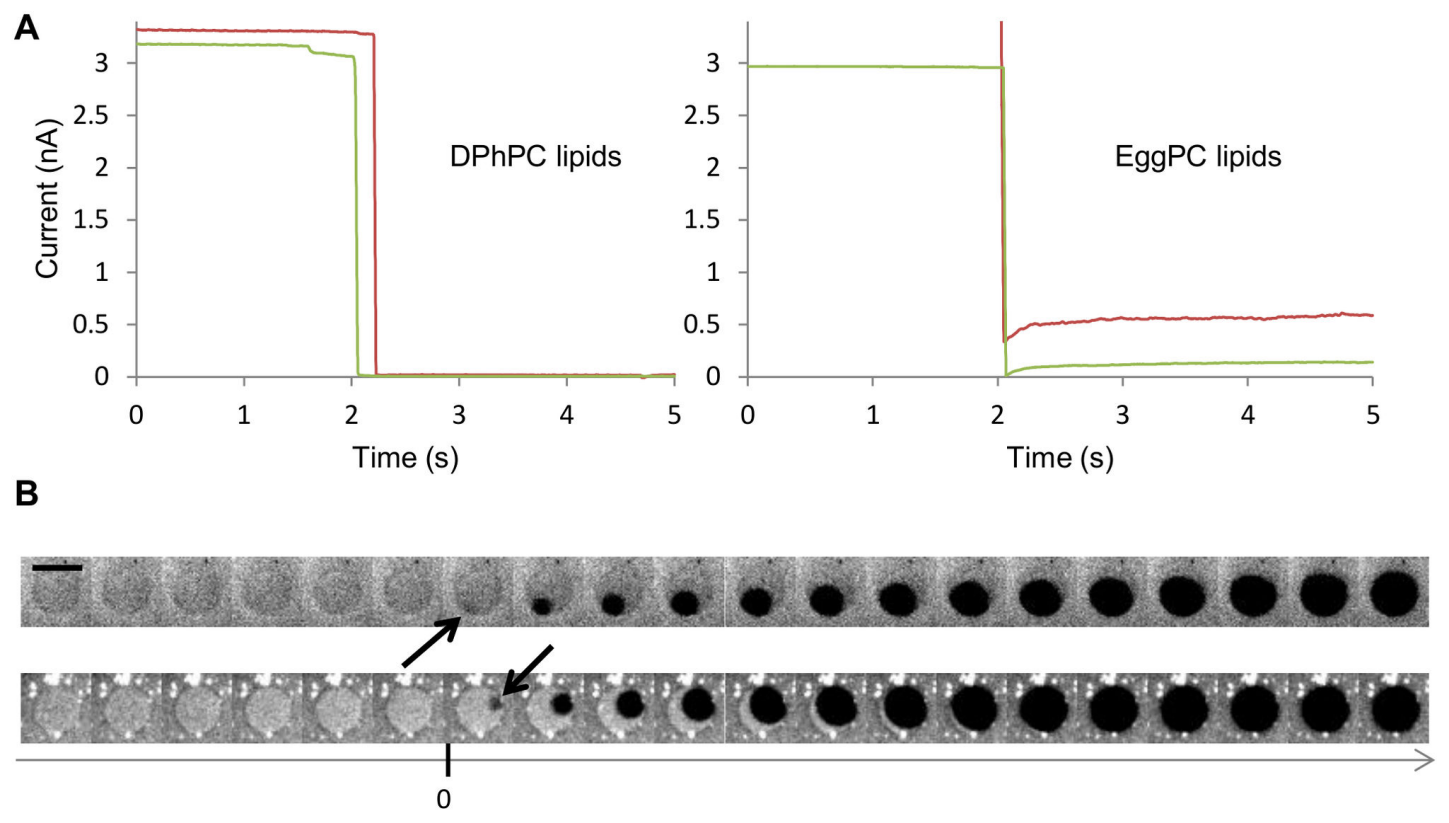
Capillary patch clamp stability. A\ Traces of ionic current during the formation of a membrane patch from DPhPC GUVs (left) and from EggPC GUVs (right). Gigaseals are obtained only for DPhPC lipids. B\ Time lapse images centered on the ID region of the capillary, showing two examples of DPhPC membrane patch breakage. For t < 0, the membrane patch is intact and visible thanks to fluorescently labeled membrane. At t = 0, holes are nucleated at the contact circle between the supported and free standing membrane region. At t > 0, the nucleated holes expand progressively until they uncover the totality of the capillary ID. Images are separated by 0.1 s. Scale bar: 10 μm.

Preformed GUVs were subsequently added to the chamber (mounted on a Z piezo stage) and accumulated on the casein-coated glass bottom due to buoyancy mismatch. A single GUV with a diameter of about 3 times the pipette aperture diameter was centered 20 µm below the pipette tip. The chamber was then slowly raised until vesicle fusion. A bilayer (partly suspended partly supported) formed and spanned over the aperture (Figure 3A and B) concomitantly with an observed current drop (Figure 3C). After the burst, the membrane presents an asymmetric shape, which was already reported as a consequence of asymmetric rupture [23]. Electrical seal quality was immediately tested and only membranes above 800 MΩ were kept. As shown in Figure 4A, EggPC (Sigma Aldrich) formed leaky membranes, generating 100 MΩ seals. DPhPC (Avanti Polar Lipids) resulted in gigasealed membranes with a rms noise amplitude of 5 pA at 1 kHz (Figure S3).

### Current and fluorescence detection of T5 bacteriophage DNA release

**Figure 5:**
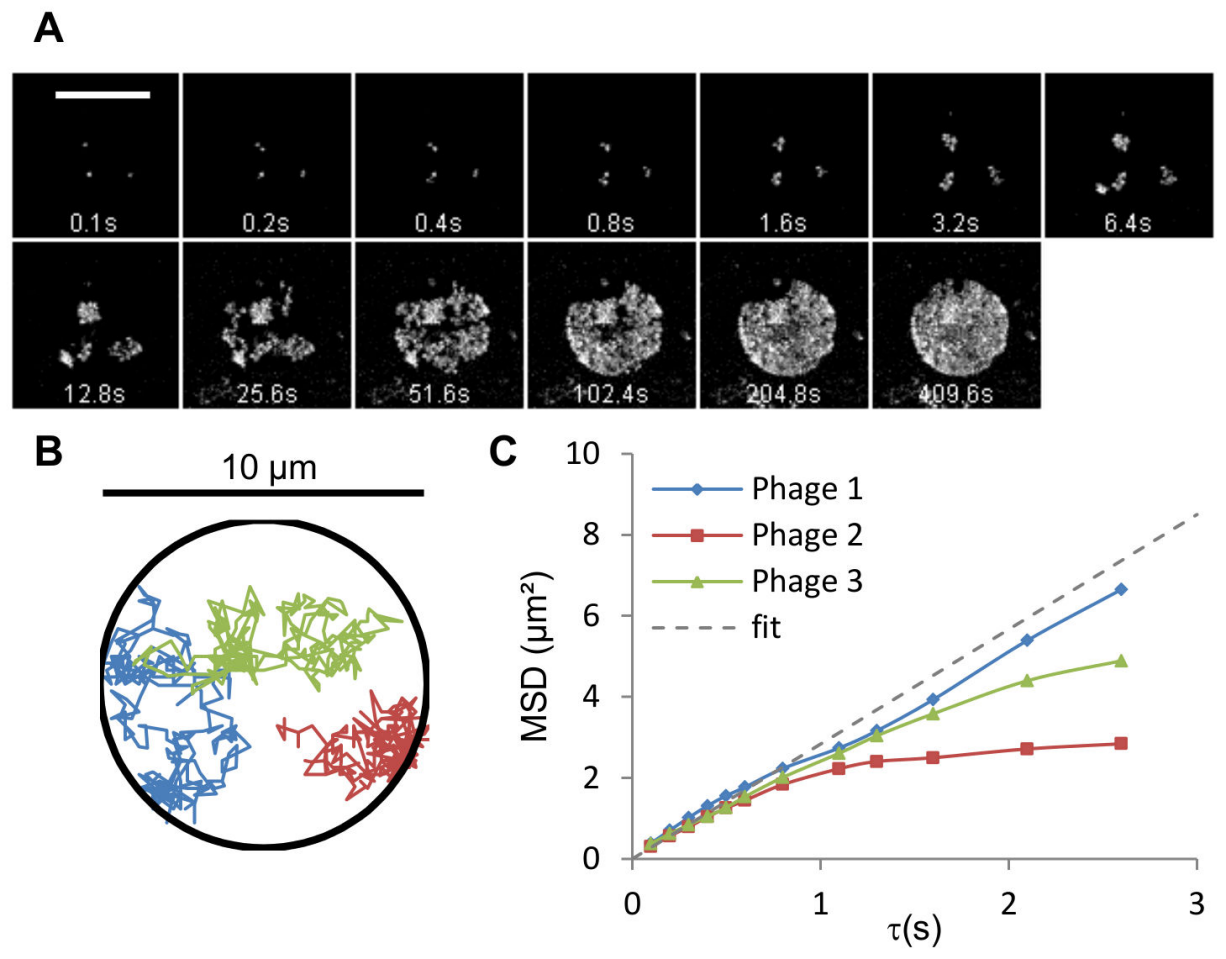
Phage T5 diffusion on lipid membrane. A\ Fluorescence image series showing diffusion of three individual T5 phages. The images are centered on the capillary ID. Three bound phages are visible on the suspended bilayer part (circular patch, 10 μm in diameter), for which intracapsid DNA is stained (Yo-Pro I). To emphasize the regions explored by the phages, a series of maximum intensity projection over time is performed; successive images correspond to a doubling time period, starting from one image (0.1 s). B\ Tracked trajectories of the 3 phages over a 10 s period. C\ Diffusion coefficient calculation for the three phages. MSD stands for mean squared displacement. The mean of the three values obtained for each phage is 2.6 μm^2^.s^-1^. Deviation from free Brownian motion (dashed line) is a consequence of constrained diffusion area (see phage n°2).

Phages with their DNA pre-stained were added to the chamber (cis side) at a concentration of 10^10^ pfu/mL. Phages bound equally to the supported part and suspended part of the lipid bilayer, provided that it was functionalized with FhuA. They remained immobile on the supported part (most probably because FhuA has long loops extending out of the membrane that are stuck to the glass surface) whereas they freely diffused in the suspended region (Figure 5A). Their diffusion coefficient (D) was assessed by individually tracking each phage particle, which appears as a dot before it ejects its DNA. Measuring the 2D mean squared displacement, we found a value for D of 2.6 µm^2^.s^-1^ (Figure 5B and C). As one phage binds to one FhuA [24], we can suppose this value corresponds to the diffusion coefficient of a phage particle bound to one FhuA protein. Both FhuA and T5 contribute significantly to this coefficient: on the one hand, we calculated that the phage particle 3D diffusion coefficient is 3.8 µm^2^.s^-1^ (approximated to a sphere, 50 nm in radius); on the other hand, transmembrane protein diffusion coefficient varies between 2 and 10 µm^2^.s^-1^, depending on the membrane composition [25].

**Figure 6:**
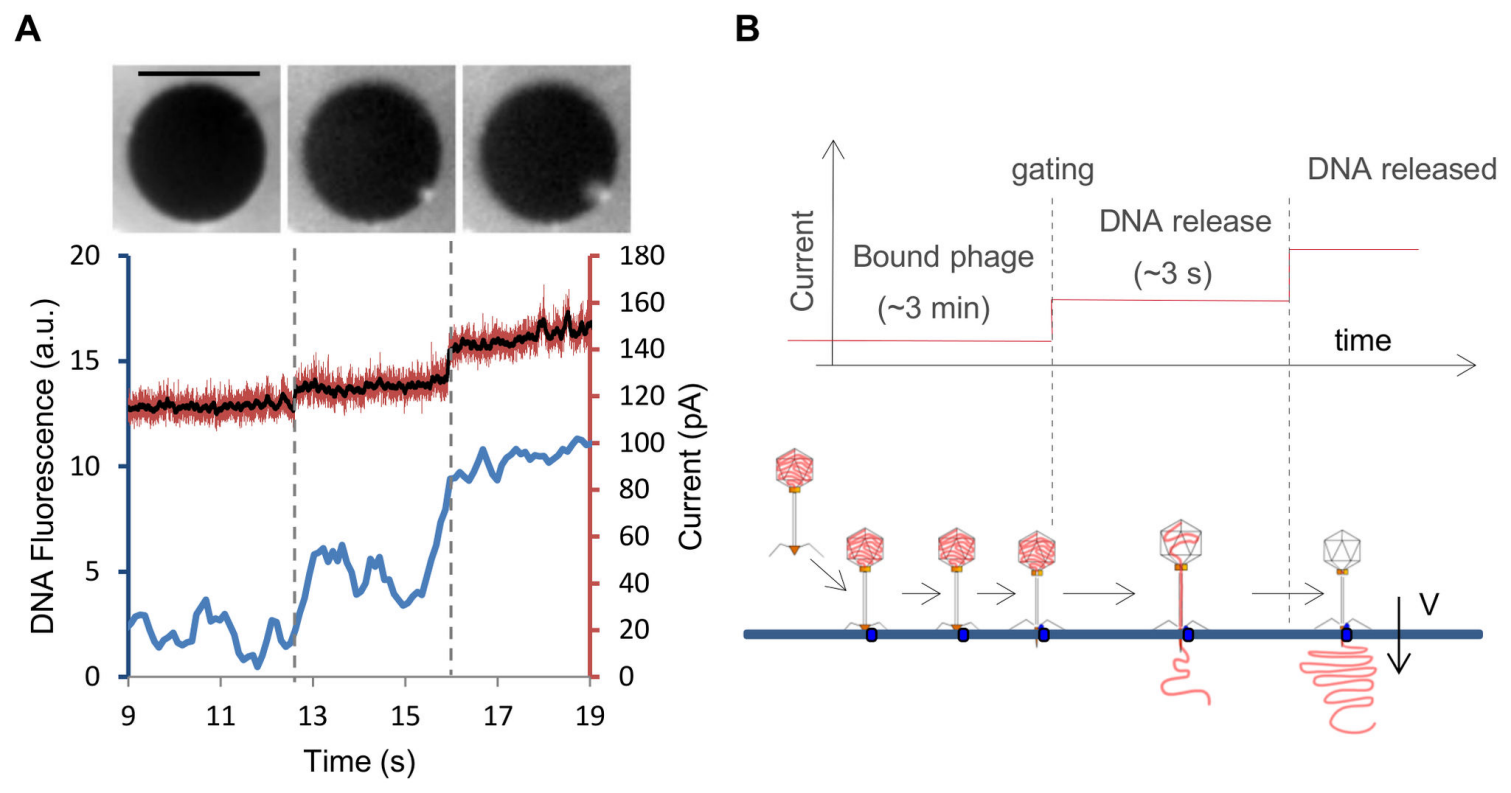
Simultaneous monitoring of T5 DNA release and phage induced pore formation through the lipid membrane. A\ Simultaneous recording of electrophysiological activity of the phage induced pore and fluorescence image of released DNA. Three phases are visible. Left (t < 12.5 s): the DNA has not been released. Middle (12.5 s < t < 16 s): fluorescence increase period i.e. DNA release step. Right (t > 16 s): fluorescence plateau reached, the DNA has been completely released. Top: mean of Yo-Pro I fluorescence images obtained for the three periods. Unstained phages were used. Hence Yo-Pro I stains only the DNA released from phages, and not the intracapsid DNA. Bottom: the amount of fluorescence increase is quantified and shown over time (blue curve). Simultaneous current measurement is shown in red (black: mean current). Two current steps flank the beginning and the end of the fluorescence increase period. Applied voltage = -20 mV. Scale bar: 10 μm. B\ Suggested sequence of events leading to phage DNA ejection. See discussion for details.

In order to further visualize DNA ejection itself, unstained phages were used to avoid pre-ejection photoinduced damage or capsid rupture. Yo-Pro I was present in the observation chamber at a 1 µM concentration, which allows immediate staining of every free DNA strand once out of the capsid but does not label intracapsid DNA over a one-hour course. It follows that binding events of phages onto the membrane could not be imaged. DNA ejection alone was visualized while transmembrane ionic current was recorded. Following N = 3 successful events, correlated signals between the transmembrane conductivity and the optical signal were obtained (Figure 6A). DNA release was followed in real time by monitoring the temporal variation of fluorescence intensity as intercalating dyes are instantaneously incorporated in the ejected dsDNA. On the example displayed in Figure 6, DNA release occurs in 3.5 s (typically 3 s) and correlates with a step increase in the transmembrane conductivity of 0.5 nS. These values are in line with [8,9]. The conductance remains constant during DNA release and then rises by another 1 nS concomitantly with the arrest of fluorescence increase.

## Discussion

### A new technique to combine fluorescence, electrophysiological measurement and mechanical stimulation on a suspended membrane

Few *in vitro* assays and studies allow the combination of both electrical measurements and imaging: previous achievements used either a membrane formed by thinning a film of lipids dissolved in organic solvents (Black Lipid Membrane or BLM), or an aqueous droplet immerged in oil and dissolved lipids which is put into close contact with a thin hydrogel [26] (Droplet on Hydrogel Bilayers or DHBs). In both cases, the presence of organic solvents prevents direct incorporation of the membrane proteins that were subsequently added after membrane formation by several non-quantitative methods (Ca^2+^ induced fusion, or membrane protein solubilization). An alternative approach consisting in patching GUVs functionalized with ion channels has already been devised [27,28]. Nonetheless, in the particular case of FhuA, it is more difficult to detect membrane protein incorporation since FhuA has no intrinsic electrical activity. Two previous studies [8,19] report reconstitution of FhuA within BLMs, however the second study underlies that they were unable to reproduce data of the first publication, emphasizing the difficulty of reconstituting active transmembrane proteins.

Our approach partly solves this issue since it provides a consistent and repeatable way of generating a FhuA functionalized bilayer. However the fraction of functional FhuA remains low. Indeed, after 1 hour of incubation no more than 1 phage per 10 µm^2^ of vesicle area was observed. Given that the FhuA/lipid weight ratio we used is 1%, we estimate a protein density of around 200 FhuA mol/µm^2^ of membrane. As the membrane is symmetrical, we expect that only half the proteins are inserted in the correct orientation. However, we find that 0 to 10% of bound phages (with an average of 2%) eject their DNA in the GUV. We checked with a bulk assay that neither labeling of FhuA molecules (Figure S1) nor FhuA reconstitution into SUVs (Figure S2) alters drastically FhuA ability to induce phage DNA release. Additionally, 90% of phages were able to eject their DNA with solubilized FhuA (data not shown). Hence we estimate that only a fraction of 0.1% of FhuA proteins are active in the reconstituted membrane, assuming a physiological 1/1 relationship between phage and FhuA (the most probable hypothesis [29]). We concluded that FhuA proteins are being inactivated during GUV formation by electroswelling, a process made at low salt concentrations, a condition known to reduce protein activity. Ideally, one should use methods that better preserve proteins to produce GUVs. Yet only electroswelling in low salt leads to a high yield of large (>20 µm) GUVs, which are needed for the method we developed. Although the total incorporation yield is low, it is still high enough to perform single phage observations on suspended bilayers of 10 µm in diameter. 

Another advantage of the technique is that it provides a way to control the density of incorporated proteins without organic solvent use. The reconstitution process can be controlled at several steps, if using fluorescently labeled proteins. It allows controlling of the membrane biochemical properties (cis/trans solution asymmetry, lipid composition, membrane proteins composition). Additionally, the membrane is much larger than in standard patch clamp experiments (5-20 µm in diameter whereas standard patch pipettes have a 1-2 µm wide diameter), which is an advantage for optical detection. 

However the method suffers from several drawbacks. The experimental setup is sophisticated and micromanipulation is required. In addition, maintaining protein activity during GUV formation is difficult, but constant progress and new techniques are made in this field [18,30–34]. Lastly membrane stability (i.e. the amount of time spent with a membrane resistance higher than 1 GΩ) varies between bilayers from a few seconds to tens of minutes (Figure S4E). We observed that membranes mainly break at the contact line between free-standing membrane and supported membrane (Figure 4B and Figure S4). Membrane stability might be improved by stabilizing the line connecting the membrane patch to the supported membrane. Another way to improve membrane stability is to use lipids with long carbon chains and a low insaturation degree, as they are less prone to break [35].

**Figure 7:**
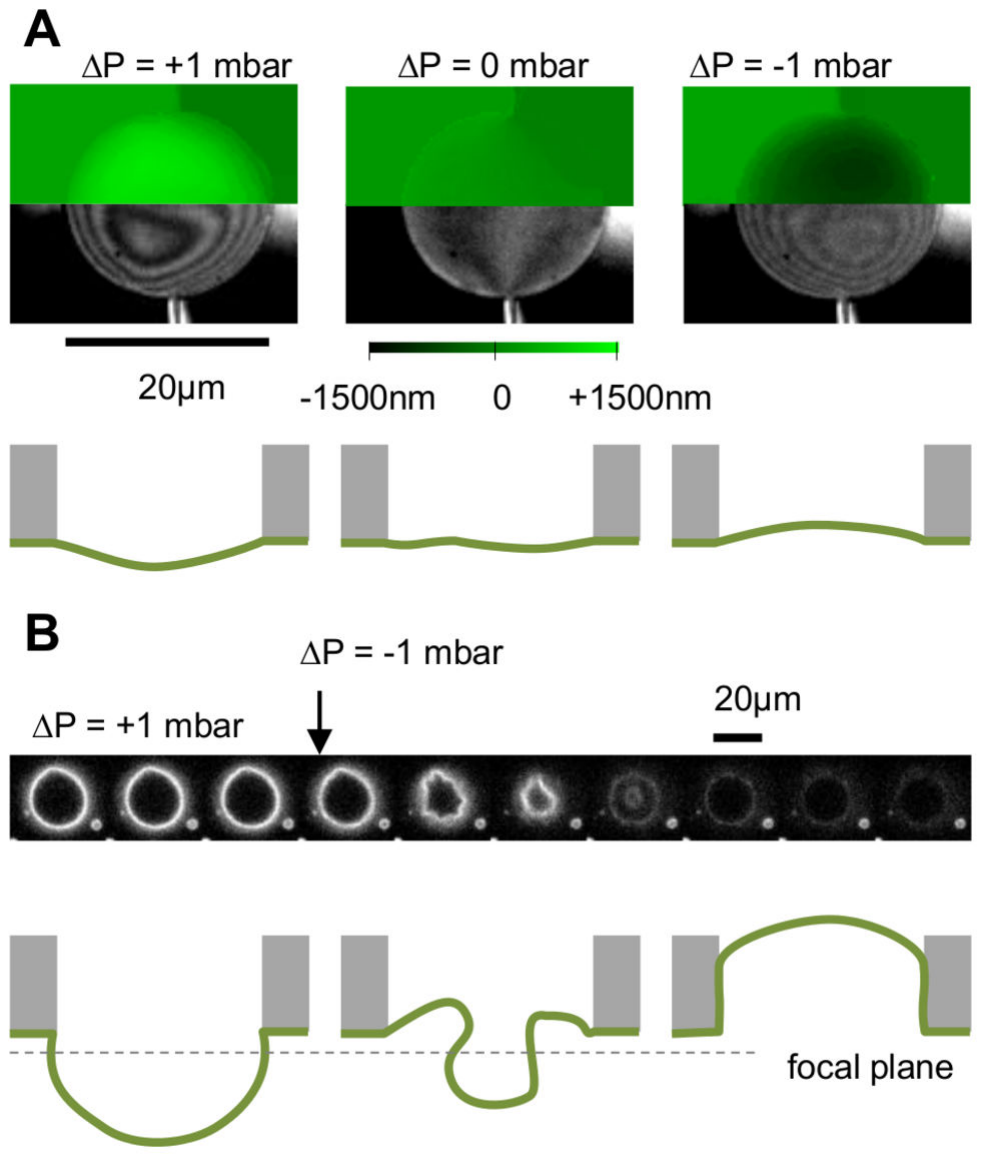
Pressure control and membrane deformation, under limited glass adhesion conditions. A\ RICM visualization of membrane deformation as response of applied pressure on membrane and corresponding sketch. B\ A large excess of membrane can sometimes be obtained. Large fluctuations can be observed at nearly equal pressure that lead to huge membrane fluctuations.

**Figure 8:**
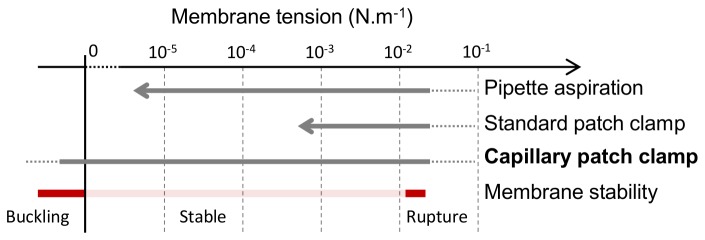
Range of accessible membrane tension. Schematic description of the accessible membrane tension range for the pipette aspiration technique, for standard patch clamp, and for our new capillary patch clamp method (if limiting glass adhesion surface). The typical regions where membranes are stable, buckling or breaking are shown in red.

Another point worth noticing is that our method can be used to obtain low tensed membrane patch, in case we passivate glass just after gigaseal formation (see Supporting Information S1). Like other methods, setting the hydrostatic pressure difference between the inside and the outside of the capillary allows us to indirectly control membrane tension. However, reaching the very low tension regime (i.e. highly fluctuating membrane) is very difficult. The reason is that most of glass patching methods artificially induce a high membrane tension set by the adhesion energy between glass and lipids [36]. Thus even at zero pressure difference between cis and trans compartment, there is a resting membrane tension which is always very high (~>10^-3^ N.m^-1^). This high tension potentially affects membrane protein function. It also leads to an “electrophysiological blind-spot”, i.e. membrane tensions for which no data are currently available, as hypothesized by the authors of [36]. In membrane mechanics studies it is possible to span a much larger range of membrane tensions by using the pipette aspiration technique. Spanning 4 orders of magnitude has been reported [37]. It remains however very difficult to set a tension below the resting tension of the held vesicle. By using our method with glass passivation (details in Supporting Information S1 and Figure S4D), the resting tension at zero pressure difference can be dramatically lowered, as it no longer depends on lipid/glass adhesion. At equal pressure between cis and trans compartment, we could observe highly fluctuating membranes, and even buckling instabilities when the excess of membrane was very large (Figure 7), a result already reported for some specific membrane compositions [38]. We did not measure tension precisely, but our capability to reach both the highly fluctuating regime and the breaking limit indicates that our method allows us to span the entire range of membrane tension (a summary of the method capabilities is presented Figure 8). We postulate that reaching very low tensions may be important to study processes such as endosomal trafficking, or to tackle mitochondria/Golgi/ER shape.

### Discussion on bacteriophage T5 DNA ejection process

Most DNA ejection studies were conducted in absence of a lipid membrane, as DNA ejection of T5 [9,17,39], lambda [21,22,40,41], SPP1 [42], 9NA and P22 [43] can be triggered *in vitro* by simple docking to their purified receptors solubilized in detergent. In this context and regarding T5 phage, DNA ejection in solution was demonstrated to occur in 3 steps: a binding step to its receptor FhuA with a high rate constant (3.10^6^ M^-1^.s^-1^), an activation step during which conformational rearrangements of the tail proteins are likely to occur over a period of around 10 min (24°C), and a DNA release step when the dsDNA is released in the surrounding medium in around 3 s [9]. Yet, the conditions of these dynamical studies [9,21,22,39] differ a lot from the *in vivo* situation where FhuA proteins are inserted in the outer membrane of *E. coli*. Other approaches including lipid bilayers already gave us clues as to the T5 DNA ejection process. For instance, cryo-EM studies have shown that the T5 phage ejection process could be reconstituted across the lipid bilayer of large unilamellar vesicles functionalized with FhuA [44,45]. Additionally, gating events in the conductivity of BLMs containing FhuA and in presence of bacteriophages have also been reported [8,19], indicative of transmembrane pore formation during DNA ejections. Single phage docking resulting in conductivity changes were also reported for the lambda phage and its membrane receptor LamB [10,11]. FhuA has extensively been studied [19,46–48], but the formation origin of a phage-induced pore remains unclear. In the case of T5, phage docking to FhuA was proposed to result in conformational changes of a protein located at the tip of the tail that would form a pore through which the DNA would be released. The exact order of the various events (pore formation by the distal tail proteins, DNA release after conformational changes of the portal protein, docking, etc.) still remains elusive.

Based on the simultaneous detection of electrophysiological signal and fluorescence imaging, our data suggest the following sequence of events for the phage DNA ejection process (Figure 6B). Phages bind to their membrane receptor with a high affinity and no noticeable conductance changes. This suggests that the docking of FhuA to the phage and the resulting protein conformational changes do not instantly lead to pore formation. Indeed the phage capsid and tail are porous to ions [49], we thus expect to obtain detectable ionic current as soon as a transmembrane pore is assembled, even at the tip of an empty tail. After docking, it takes 3 to 10 minutes at 25°C to observe concomitant ejection of DNA and pore formation. It suggests that the portal protein complex that gates the DNA passage from the capsid into the hollow tail opens concomitantly or prior to the pore formed at the tail tip. Pore formation is thus the final step of the activation phase. One could even hypothesize that the intra-tail pressure increase due to DNA passage could be the triggering event of pore formation. During the entire DNA release step, the conductance remains constant. We believe it reflects the conductance of the pore with dsDNA in it. The conductance abruptly changes at the end of ejection, most likely revealing the conductance of the empty tail linked to the empty capsid. However, DNA is still attached to the phage/FhuA complex. Indeed, using our first GUV observations, we noticed that the DNA strand remains attached to the phage/FhuA complex (Figure 1C) for at least 2 h at least after the end of the ejection. Due to the 2D diffusion of the bacteriophage particles on the vesicle surface, it is hard to determine the exact length of ejected DNA. However, based on the lateral extension of the DNA fluctuations (~2 µm) and based on the comparison with images taken for fully ejected DNA, we can conclude that the DNA is almost entirely ejected. The abrupt conductance change confirms that the fraction of DNA still bound is very small and could be loosely bound to the pore complex. The final attachment of DNA to its capsid has already been observed in various circumstances where phages were adsorbed on solid substrates [9,21,22]. However it was speculated that it could be an artifact of non-specific surface adhesion at the DNA end. In our system this hypothesis is ruled out and we demonstrate that the attachment really occurs via the phage/FhuA complex.

## Conclusions

In conclusion, we propose an innovative method to create suspended membrane bilayers that can be imaged using high resolution microscopy while being monitored for their single ion channel activity. We used a two-step process comprising of the reconstitution of membrane protein in GUV followed by the formation of the bilayer by GUV fusion over the tip of a capillary tube. This method offers the possibility to reconstitute transmembrane proteins into suspended bilayer of controlled tension. Simultaneous imaging and electrophysiological measurements could be achieved. We illustrate the use of our method by studying the *in vitro* reconstitution of DNA ejection from a T5 bacteriophage.

## Supporting Information

Supporting Information S1 **Detailed method: capillary patch clamp with glass passivation.**
(DOCX)

Figure S1 **Control of labeled FhuA activity.** A\ Following the protocol published in (5), we tested the activity of Alexa labeled FhuA (dashed curve) vs. unlabelled FhuA (continuous curve). The amount of ejected DNA was measured as a function of time with 1 µm Yo-Pro I with a spectrofluorometer at 37°C. FhuA was added at (t = 0) at 40 nM in both cases, in a regime where the kinetics highly depends on FhuA concentration. The curves were both normalized to the final fluorescence plateau, a state corresponding to all phages having ejected their DNA. The good overlay of curves proves that FhuA labeling does not impair its activity.
(TIF)

Figure S2 **Control of FhuA activity in reconstituted SUVs.** As explained in materials and methods, DPhPC-FhuA SUVs are recovered by centrifugation after dialysis. In this figure, the ability to induce DNA phage ejection is tested for: a – the recovered DPhPC-FhuA SUV pellet (squares), b – its supernatant (continuous gray line), c – positive control (solubilized FhuA not inserted in SUVs) (dashed line), d – negative control (triangles). The kinetics is similar between positive control and the DPhPC-FhuA SUV pellet. An offset is clearly visible due to lipid induced fluorescence.
(TIF)

Figure S3 **Gigaseal current example.** A\ The membrane patch is formed at t = 0 s; 20 mV are applied. B and C\ Zoom on the signal 10 s and 30 s after patch formation. Evenly spaced peaks can be seen, that correspond to electrical leak from the camera acquiring at 10 fps.
(TIF)

Figure S4 **Membrane behavior with or without glass passivation.** A-B-C\ No glass passivation. A\ Sketch showing membrane behavior upon membrane break: the suspended part disappears and the corresponding lost surface is recovered at the supported membrane edges. B\ Experimental data showing the dynamics of suspended membrane disappearance upon membrane break (red) and corresponding supported membrane surface increase (green). C\ Left: patched GUV on a capillary before the suspended membrane break. Middle: final state of the membrane after rupture. Right: Subtraction of the middle image from the left image. A lookup table is applied to show the negative values in red (membrane that has disappeared), and the positive values in green (membrane that has appeared). Colors are corresponding with B. D\ With glass passivation. Left: sketch showing the two passive glass surfaces: the inside of the capillary and the surface around the supported membrane. Right: putative model of membrane break in the low tension regime: a stable nanohole is nucleated at the edge of the suspended membrane. The nanohole remains below optical resolution but the gigaseal is lost. E\ Membrane patch gigaseal lifetime observed for non passivated glass surface (left, N = 34) and for passivated glass surface (right, N = 9).
(TIF)

## References

[1] Hamill OP, Marty A, Neher E, Sakmann B, Sigworth FJ (1981) Improved patch-clamp techniques for high-resolution current recording from cells and cell-free membrane patches. Pflugers Arch 391: 85–100. doi:10.1007/BF00656997. PubMed: 6270629. 6270629

[2] Kasianowicz JJ, Brandin E, Branton D, Deamer DW (1996) Characterization of individual polynucleotide molecules using a membrane channel. Proc Natl Acad Sci U S A 93: 13770–13773. doi:10.1073/pnas.93.24.13770. PubMed: 8943010. 8943010 PMC19421

[3] Robertson JWF, Rodrigues CG, Stanford VM, Rubinson KA, Krasilnikov OV et al. (2007) Single-molecule mass spectrometry in solution using a solitary nanopore. Proc Natl Acad Sci U S A 104: 8207–8211. doi:10.1073/pnas.0611085104. PubMed: 17494764. 17494764 PMC1866312

[4] Venkatesan BM, Bashir R (2011) Nanopore sensors for nucleic acid analysis. Nat Nanotechnol 6: 615–624. doi:10.1038/nnano.2011.129. PubMed: 21926981. 21926981

[5] Kodandaramaiah SB, Franzesi GT, Chow BY, Boyden ES, Forest CR (2012) Automated whole-cell patch-clamp electrophysiology of neurons in vivo. Nat Methods 9: 585–587. doi:10.1038/nmeth.1993. PubMed: 22561988. 22561988 PMC3427788

[6] Borisenko V, Lougheed T, Hesse J, Füreder-Kitzmüller E, Fertig N et al. (2003) Simultaneous optical and electrical recording of single gramicidin channels. Biophys J 84: 612–622. doi:10.1016/S0006-3495(03)74881-4. PubMed: 12524314. 12524314 PMC1302642

[7] Honigmann A, Walter C, Erdmann F, Eggeling C, Wagner R (2010) Characterization of horizontal lipid bilayers as a model system to study lipid phase separation. Biophys J 98: 2886–2894. doi:10.1016/j.bpj.2010.03.033. PubMed: 20550901. 20550901 PMC2884238

[8] Bonhivers M, Ghazi A, Boulanger P, Letellier L (1996) FhuA, a transporter of the Escherichia coli outer membrane, is converted into a channel upon binding of bacteriophage T5. EMBO J 15: 1850–1856. PubMed: 8617231. 8617231 PMC450102

[9] Chiaruttini N, de Frutos M, Augarde E, Boulanger P, Letellier L et al. (2010) Is the In Vitro Ejection of Bacteriophage DNA Quasistatic? A Bulk to Single Virus Study. Biophys J 99: 447–455. doi:10.1016/j.bpj.2010.04.048. PubMed: 20643062. 20643062 PMC2905079

[10] Berrier C, Bonhivers M, Letellier L, Ghazi A (2000) High-conductance channel induced by the interaction of phage lambda with its receptor maltoporin. FEBS Lett 476: 129–133. doi:10.1016/S0014-5793(00)01705-1. PubMed: 10913599. 10913599

[11] Gurnev PA, Oppenheim AB, Winterhalter M, Bezrukov SM (2006) Docking of a single phage lambda to its membrane receptor maltoporin as a time-resolved event. J Mol Biol 359: 1447–1455. doi:10.1016/j.jmb.2006.04.034. PubMed: 16697410. 16697410

[12] Letellier L, Plançon L, Bonhivers M, Boulanger P (1999) Phage DNA transport across membranes. Res Microbiol 150: 499–505. doi:10.1016/S0923-2508(99)00107-2. PubMed: 10577483. 10577483

[13] Bertin A, de Frutos M, Letellier L (2011) Bacteriophage-host interactions leading to genome internalization. Curr Opin Microbiol 14: 492–496. doi:10.1016/j.mib.2011.07.010. PubMed: 21783404. 21783404

[14] Ide T, Takeuchi Y, Yanagida T (2002) Development of an Experimental Apparatus for Simultaneous Observation of Optical and Electrical Signals from Single Ion Channels. Single Mol 3: 33–42. doi:10.1002/1438-5171(200204)3:1.

[15] Bayley H, Cronin B, Heron A, Holden MA, Hwang WL et al. (2008) Droplet interface bilayers. Mol Biosyst 4: 1191–1208. doi:10.1039/b808893d. PubMed: 19396383. 19396383 PMC2763081

[16] Sonnleitner A, Mannuzzu LM, Terakawa S, Isacoff EY (2002) Structural rearrangements in single ion channels detected optically in living cells. Proc Natl Acad Sci U S A 99: 12759–12764. doi:10.1073/pnas.192261499. PubMed: 12228726. 12228726 PMC130533

[17] Boulanger P, le Maire M, Bonhivers M, Dubois S, Desmadril M et al. (1996) Purification and Structural and Functional Characterization of FhuA, a Transporter of the Escherichia coli Outer. Membrane. Biochemistry 35: 14216–14224. 10.1021/bi9608673 8916906

[18] Girard P, Pécréaux J, Lenoir G, Falson P, Rigaud J-L et al. (2004) A new method for the reconstitution of membrane proteins into giant unilamellar vesicles. Biophys J 87: 419–429. doi:10.1529/biophysj.104.040360. PubMed: 15240476. 15240476 PMC1304363

[19] Udho E, Jakes KS, Buchanan SK, James KJ, Jiang X et al. (2009) Reconstitution of bacterial outer membrane TonB-dependent transporters in planar lipid bilayer membranes. Proc Natl Acad Sci U S A 106: 21990–21995. doi:10.1073/pnas.0910023106. PubMed: 19959664. 19959664 PMC2799797

[20] Lambert O, Plançon L, Rigaud JL, Letellier L (1998) Protein-mediated DNA transfer into liposomes. Mol Microbiol 30: 761–765. doi:10.1046/j.1365-2958.1998.01107.x. PubMed: 10094624. 10094624

[21] Grayson P, Han L, Winther T, Phillips R (2007) Real-time observations of single bacteriophage lambda DNA ejections in vitro. Proc Natl Acad Sci U S A 104: 14652–14657. doi:10.1073/pnas.0703274104. PubMed: 17804798. 17804798 PMC1976217

[22] Wu D, Van Valen D, Hu Q, Phillips R, Van Valen D et al. (2010) Ion-dependent dynamics of DNA ejections for bacteriophage lambda. Biophys J 99: 1101–1109. doi:10.1016/j.bpj.2010.06.024. PubMed: 20712993. 20712993 PMC2920739

[23] Hamai C, Cremer PS, Musser SM (2007) Single giant vesicle rupture events reveal multiple mechanisms of glass-supported bilayer formation. Biophys J 92: 1988–1999. doi:10.1529/biophysj.106.093831. PubMed: 17189305. 17189305 PMC1861791

[24] Plançon L, Janmot C, Le Maire M, Desmadril M, Bonhivers M et al. (2002) Characterization of a high-affinity complex between the bacterial outer membrane protein FhuA and the phage T5 protein pb5. J Mol Biol 318: 557–569. doi:10.1016/S0022-2836(02)00089-X. PubMed: 12051859. 12051859

[25] Ramadurai S, Holt A, Krasnikov V, van den Bogaart G, Killian JA et al. (2009) Lateral diffusion of membrane proteins. J Am Chem Soc 131: 12650–12656. doi:10.1021/ja902853g. PubMed: 19673517. 19673517

[26] Heron AJ, Thompson JR, Mason AE, Wallace MI (2007) Direct detection of membrane channels from gels using water-in-oil droplet bilayers. J Am Chem Soc 129: 16042–16047. doi:10.1021/ja075715h. PubMed: 18052065. 18052065

[27] Aimon S, Manzi J, Schmidt D, Poveda Larrosa JA, Bassereau P et al. (2011) Functional reconstitution of a voltage-gated potassium channel in giant unilamellar vesicles. PLOS ONE 6: e25529. doi:10.1371/journal.pone.0025529. PubMed: 21998666. 21998666 PMC3188570

[28] Gornall JL, Mahendran KR, Pambos OJ, Steinbock LJ, Otto O et al. (2011) Simple reconstitution of protein pores in nano lipid bilayers. Nano Lett 11: 3334–3340. doi:10.1021/nl201707d. PubMed: 21749149. 21749149

[29] Flayhan A, Wien F, Paternostre M, Boulanger P, Breyton C (2012) New insights into pb5, the receptor binding protein of bacteriophage T5, and its interaction with its Escherichiacoli receptor FhuA. Biochimie 94: 1989–1982. 10.1016/j.biochi.2012.05.021 22659573

[30] Varnier A, Kermarrec F, Blesneac I, Moreau C, Liguori L et al. (2010) A simple method for the reconstitution of membrane proteins into giant unilamellar vesicles. J Membr Biol 233: 85–92. doi:10.1007/s00232-010-9227-8. PubMed: 20135103. 20135103

[31] Richmond DL, Schmid EM, Martens S, Stachowiak JC, Liska N et al. (2011) Forming giant vesicles with controlled membrane composition, asymmetry, and contents. Proc Natl Acad Sci U S A 108: 9431–9436. doi:10.1073/pnas.1016410108. PubMed: 21593410. 21593410 PMC3111313

[32] Montes LR, Alonso A, Bagatolli LA (2007) Giant unilamellar vesicles electroformed from native membranes and organic lipid mixtures under physiological conditions. Biophys J 93: 3548–3554. doi:10.1529/biophysj.107.116228. PubMed: 17704162. 17704162 PMC2072068

[33] Pott T, Bouvrais H, Méléard P (2008) Giant unilamellar vesicle formation under physiologically relevant conditions. Chem Phys Lipids 154: 115–119. doi:10.1016/j.chemphyslip.2008.03.008. PubMed: 18405664. 18405664

[34] Dezi M, Di Cicco A, Bassereau P, Lévy D (2013) Detergent-mediated incorporation of transmembrane proteins in giant unilamellar vesicles with controlled physiological contents. Proc Natl Acad Sci U S A 110: 7276–7281. doi:10.1073/pnas.1303857110. PubMed: 23589883. 23589883 PMC3645586

[35] Evans E, Heinrich V, Ludwig F, Rawicz W (2003) Dynamic tension spectroscopy and strength of biomembranes. Biophys J 85: 2342–2350. doi:10.1016/S0006-3495(03)74658-X. PubMed: 14507698. 14507698 PMC1303459

[36] Ursell T, Agrawal A, Phillips R (2011) Lipid bilayer mechanics in a pipette with glass-bilayer adhesion. Biophys J 101: 1913–1920. doi:10.1016/j.bpj.2011.08.057. PubMed: 22004745. 22004745 PMC3192956

[37] Evans E, Rawicz W (1990) Entropy-driven tension and bending elasticity in condensed-fluid membranes. Phys Rev Lett 64: 2094–2097. doi:10.1103/PhysRevLett.64.2094. PubMed: 10041575. 10041575

[38] Bernard AAL, Guedeau-Boudeville M-A, Jullien L, di Meglio JM (2002) Raspberry vesicles. Biochim Biophys Acta-- Biomembranes 1567: 1–5. doi:10.1016/S0005-2736(02)00617-X. PubMed: 12488031. 12488031

[39] Mangenot S, Hochrein M, Rädler J, Letellier L (2005) Real-time imaging of DNA ejection from single phage particles. Curr Biol 15: 430–435. doi:10.1016/j.cub.2004.12.080. PubMed: 15753037. 15753037

[40] Novick SL, Baldeschwieler JD (1988) Fluorescence measurement of the kinetics of DNA injection by bacteriophage lambda into liposomes. Biochemistry 27: 7919–7924. doi:10.1021/bi00420a050. PubMed: 2974726. 2974726

[41] Van Valen D, Wu D, Chen YJ, Tuson H, Wiggins P et al. (2012) A Single-Molecule Hershey-Chase Experiment. Curr Biol 22: 1339–1343. doi:10.1016/j.cub.2012.05.023. PubMed: 22727695. 22727695 PMC3462812

[42] São-José C, Lhuillier S, Lurz R, Melki R, Lepault J et al. (2006) The ectodomain of the viral receptor YueB forms a fiber that triggers ejection of bacteriophage SPP1. DNA - J Biol Chem 281: 11464–11470. 16481324 10.1074/jbc.M513625200

[43] Andres D, Roske Y, Doering C, Heinemann U, Seckler R et al. (2012) Tail morphology controls DNA release in two Salmonella phages with one lipopolysaccharide receptor recognition system. Mol Microbiol 83: 1244–1253. doi:10.1111/j.1365-2958.2012.08006.x. PubMed: 22364412. 22364412

[44] Plançon L, Chami M, Letellier L (1997) Reconstitution of FhuA, an Escherichia coli outer membrane protein, into liposomes. J Biol Chem 272: 16868–16872. doi:10.1074/jbc.272.27.16868. PubMed: 9201994. 9201994

[45] Böhm J, Lambert O, Frangakis AS, Letellier L, Baumeister W et al. (2001) FhuA-mediated phage genome transfer into liposomes: a cryo-electron tomography study. Curr Biol 11: 1168–1175. doi:10.1016/S0960-9822(01)00349-9. PubMed: 11516947. 11516947

[46] Mohammad MM, Howard KR, Movileanu L (2011) Redesign of a plugged beta-barrel membrane protein. J Biol Chem 286: 8000–8013. doi:10.1074/jbc.M110.197723. PubMed: 21189254. 21189254 PMC3048687

[47] Udho E, Jakes KS, Finkelstein A (2012) TonB-Dependent Transporter FhuA in Planar Lipid Bilayers: Partial Exit of Its Plug from the Barrel. Biochemistry 51: 6753–6759. doi:10.1021/bi300493u. PubMed: 22846061. 22846061 PMC3448877

[48] Mohammad MM, Iyer R (2012) Engineering a rigid protein tunnel for biomolecular detection. J Am Chem Soc 134: 9521–9531. doi:10.1021/ja3043646. PubMed: 22577864. 22577864 PMC3415594

[49] Eriksson M, Härdelin M, Larsson A, Bergenholtz J, Akerman B (2007) Binding of intercalating and groove-binding cyanine dyes to bacteriophage T5. J Phys Chem B 111: 1139–1148. doi:10.1021/jp064322m. PubMed: 17266268. 17266268

