# Correction: A Novel Method to Couple Electrophysiological Measurements and Fluorescence Imaging of Suspended Lipid Membranes: The Example of T5 Bacteriophage DNA Ejection

**DOI:** 10.1371/annotation/a733d1cb-96c1-4125-8060-f3d9143e964b

**Published:** 2014-01-13

**Authors:** Nicolas Chiaruttini, Lucienne Letellier, Virgile Viasnoff

The affiliations of the last author are incorrect. Virgilie Viasnoff is only affiliated with institutions 1 and 4, and not institutions 2 and 3.

