# Supplementary material for: A Novel Method to Couple Electrophysiological Measurements and Fluorescence Imaging of Suspended Lipid Membranes: The Example of T5 Bacteriophage DNA Ejection
Source: PLoS One. 2013 Dec 23;8(12):e84376. doi: 10.1371/journal.pone.0084376 (PMC3871697; doi:10.1371/journal.pone.0084376)
Supplement: Supporting Information S1 — Detailed method: capillary patch clamp with glass passivation. (DOCX) [file pone.0084376.s001.docx]

# Detailed method: capillary patch clamp with glass passivation.

In our experiments, we observe that immediately after bursting, there is a short period of time (few seconds) during which the apparent membrane surface increases. This extension is due to high adhesion energy between membrane and glass. This also explains why, if a hole is nucleated in the membrane patch, the hole extends until the suspended membrane completely disappears as in figure 4B: it is pulled by the edge of the supported membrane. Accordingly, the surface lost within the internal diameter of the capillary is restored at the edge of the supported bilayer (supplementary figure S4A-B-C).

The capillary patch method we developed offers a unique possibility to limit the adhesion between glass and membrane (supplementary figure S4D). Practically, we inject in bulk casein at 1mg/ml just after the vesicle burst to passivate the glass around the supported membrane. Also the capillary used to make the pipette has to be pre-passivated with casein to avoid membrane adhesion into the pipette. With these modifications, membrane tension is no longer influenced by glass adhesion energy. In particular, at equal pressure between cis and trans compartment the suspended membrane can be highly fluctuating, indicative of a very low tensed membrane. This never happens without glass passivation. Applying a positive or a negative pressure leads to significant membrane rounding inside or outside the pipette (figure 7). The area of membrane within the suspended part varies between experiments. In figure 7 the upper part shows a situation with a small membrane reservoir, and rounding is monitored with reflection interference contrast microscopy (RICM). The lower case shows a situation with a bigger reservoir. Huge membrane fluctuations can transiently be seen when the pressure switches rapidly from positive to negative value. We did not measure precisely the membrane tension. In theory it is possible to compute it by using Laplace’s law: the radius of curvature can be measured by imaging while the difference of pressure is set by the MFCS machine.

We investigated the role of membrane tension on membrane patch stability, with the hope that a membrane under low tension (passivated with casein and at equal pressure) would keep its insulating properties much longer. That was unfortunately not the case. The gigaseal lifetime remains highly variable and do no change dramatically under low tension (supplementary figure S4E). However the observed breaking behaviors were very different. As explained before, under high tension (i.e. no passivation) losing the gigaseal is concomitant with the formation of a clear visible hole nucleated at the border between the suspended and the supported membrane (figure 4B). The current increases sharply to its value without patch. Under low tension, losing the gigaseal happens by a slow increase in current leakage. However, at the optical scale, no difference can be detected. These observations can be interpreted as follow: independently of the tension, a nanohole can nucleate at the frontier between the supported membrane and the suspended membrane, leading to gigaseal loss. This is consistent with the general rule stating that defaults are usually nucleated at interfaces (here a line connecting the suspended bilayer to the supported membrane). If tension is high, this hole is not stable: it rapidly expands leading to microscopic observations and to a sharp current increase. If tension is low, this hole is stable and remains below the optical resolution (sketch in supplementary figure S4D). This interpretation is consistent with previous models of membrane breakage [1]. The consecutive slow current increase can be attributed either to new defaults being nucleated or to slow enlargement of nanoholes.

In our study, phage diffusion imaging was performed with casein passivation at low tension to ensure long imaging, whereas simultaneous current and optical monitoring was done under high tension (no passivation), to ensure that observed current steps are not due to nucleation of stable nanoholes.

1. Evans E, Heinrich V, Ludwig F, Rawicz W (2003) Dynamic tension spectroscopy and strength of biomembranes. Biophys J 85: 2342–2350.
