# Supplementary figures and images for: A Novel Method to Couple Electrophysiological Measurements and Fluorescence Imaging of Suspended Lipid Membranes: The Example of T5 Bacteriophage DNA Ejection
Source: PLoS One. 2013 Dec 23;8(12):e84376. doi: 10.1371/journal.pone.0084376 (PMC3871697; doi:10.1371/journal.pone.0084376)

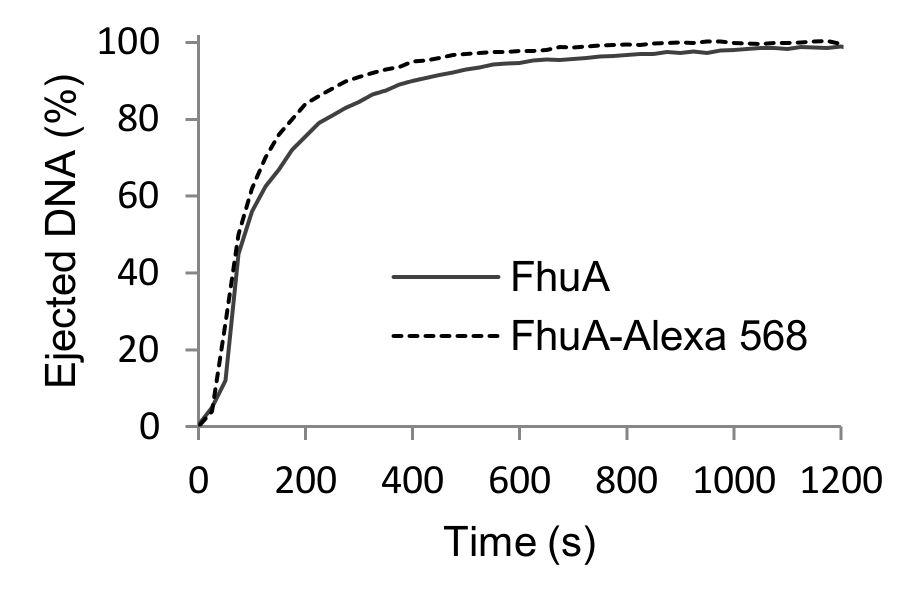

Supplement: Figure S1 — Control of labeled FhuA activity. A\ Following the protocol published in (5), we tested the activity of Alexa labeled FhuA (dashed curve) vs. unlabelled FhuA (continuous curve). The amount of ejected DNA was measured as a function of time with 1 µm Yo-Pro I with a spectrofluorometer at 37°C. FhuA was added at (t = 0) at 40 nM in both cases, in a regime where the kinetics highly depends on FhuA concentration. The curves were both normalized to the final fluorescence plateau, a state corresponding to all phages having ejected their DNA. The good overlay of curves proves that FhuA labeling does not impair its activity. (TIF) [file pone.0084376.s002.tif]

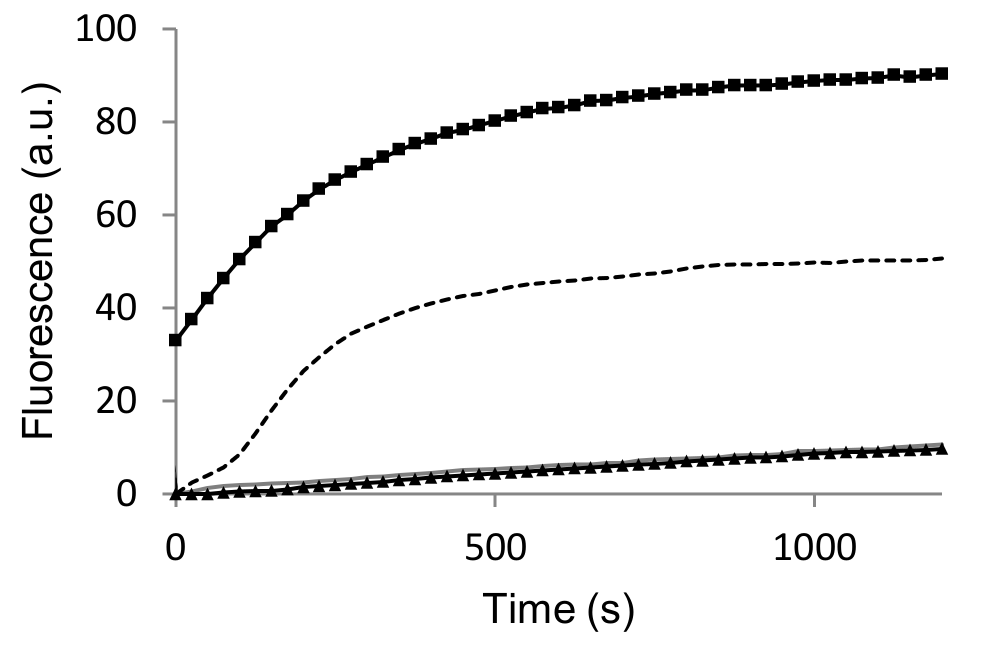

Supplement: Figure S2 — Control of FhuA activity in reconstituted SUVs. As explained in materials and methods, DPhPC-FhuA SUVs are recovered by centrifugation after dialysis. In this figure, the ability to induce DNA phage ejection is tested for: a – the recovered DPhPC-FhuA SUV pellet (squares), b – its supernatant (continuous gray line), c – positive control (solubilized FhuA not inserted in SUVs) (dashed line), d – negative control (triangles). The kinetics is similar between positive control and the DPhPC-FhuA SUV pellet. An offset is clearly visible due to lipid induced fluorescence. (TIF) [file pone.0084376.s003.tif]

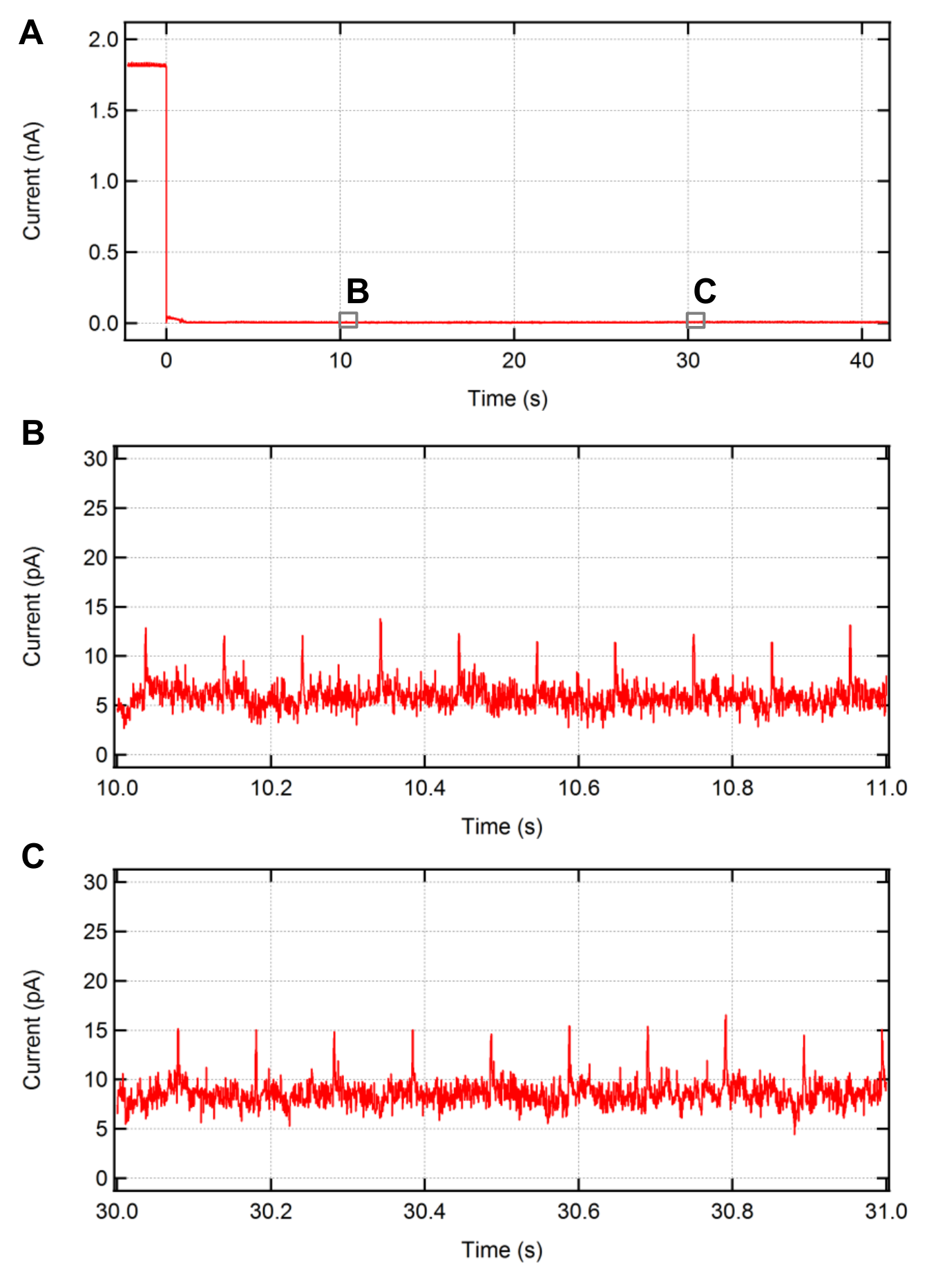

Supplement: Figure S3 — Gigaseal current example. A\ The membrane patch is formed at t = 0 s; 20 mV are applied. B and C\ Zoom on the signal 10 s and 30 s after patch formation. Evenly spaced peaks can be seen, that correspond to electrical leak from the camera acquiring at 10 fps. (TIF) [file pone.0084376.s004.tif]

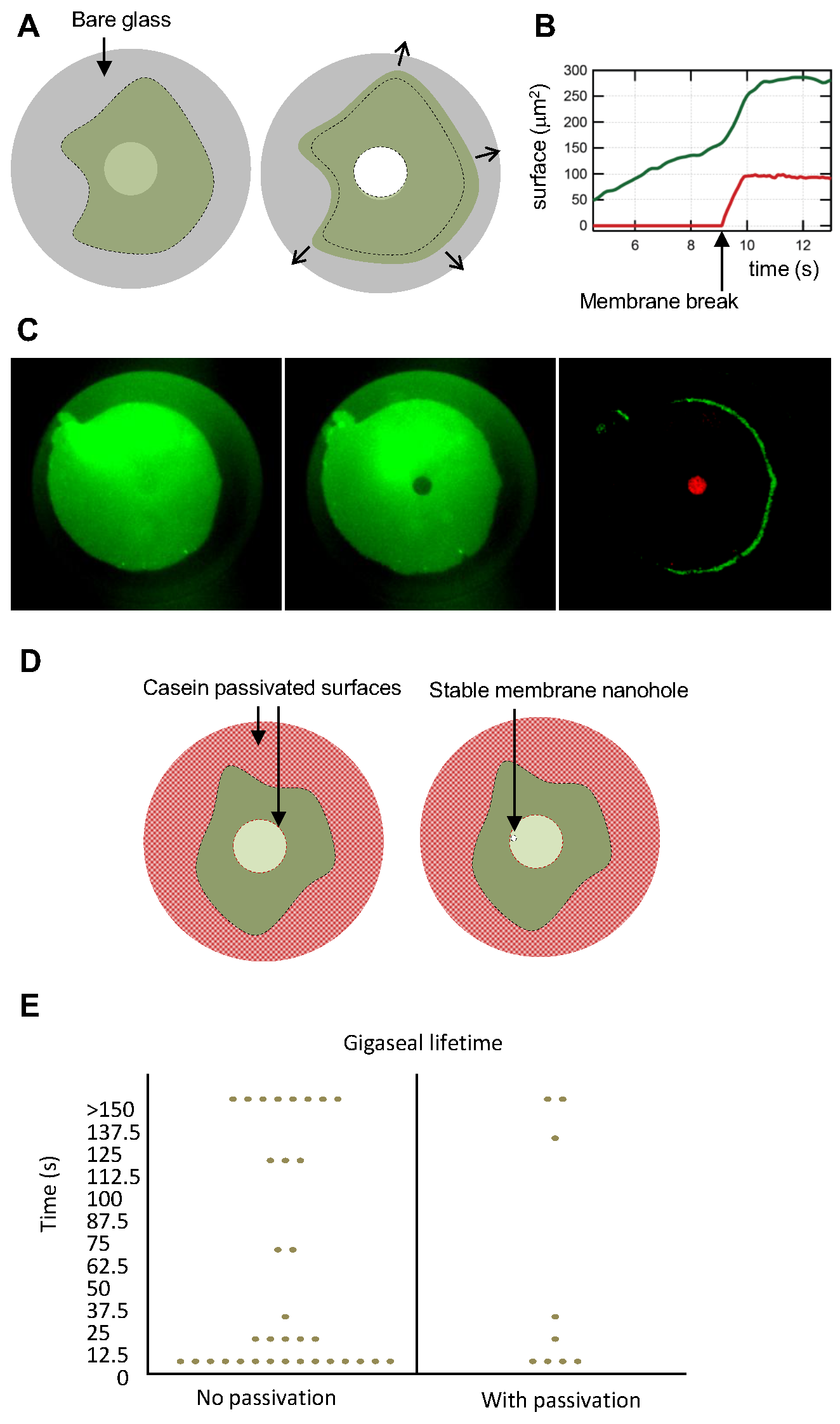

Supplement: Figure S4 — Membrane behavior with or without glass passivation. A-B-C\ No glass passivation. A\ Sketch showing membrane behavior upon membrane break: the suspended part disappears and the corresponding lost surface is recovered at the supported membrane edges. B\ Experimental data showing the dynamics of suspended membrane disappearance upon membrane break (red) and corresponding supported membrane surface increase (green). C\ Left: patched GUV on a capillary before the suspended membrane break. Middle: final state of the membrane after rupture. Right: Subtraction of the middle image from the left image. A lookup table is applied to show the negative values in red (membrane that has disappeared), and the positive values in green (membrane that has appeared). Colors are corresponding with B. D\ With glass passivation. Left: sketch showing the two passive glass surfaces: the inside of the capillary and the surface around the supported membrane. Right: putative model of membrane break in the low tension regime: a stable nanohole is nucleated at the edge of the suspended membrane. The nanohole remains below optical resolution but the gigaseal is lost. E\ Membrane patch gigaseal lifetime observed for non passivated glass surface (left, N = 34) and for passivated glass surface (right, N = 9). (TIF) [file pone.0084376.s005.tif]
